# More vasopressors were required during anesthesia maintenance with sevoflurane than remimazolam in a high-risk patient: a case report

**DOI:** 10.1186/s40981-026-00874-4

**Published:** 2026-08-18

**Authors:** Yusuke Arai, Miku Tsukamoto, Kenichi Masui

**Affiliations:** Department of Anesthesiology, Yokohama City University School of Medicine, 3-9 Fukuura, Kanazawa-Ku, Yokohama, 236-0004 Japan

**Keywords:** Remimazolam, Sevoflurane, Hypotension, Vasopressor, Phenylephrine, Noradrenaline, Case report

## Abstract

**Background:**

Remimazolam besilate is known to cause less hypotension than other anesthetics. We experienced an anesthetic switch from remimazolam to sevoflurane in a high-risk patient, allowing us to compare the intra-individual hemodynamic effects of the two anesthetics.

**Case presentation:**

A 57-year-old woman with multiple cardiovascular complications underwent a lower-limb amputation. We selected remimazolam anesthesia with peripheral nerve blocks to minimize the risk of intraoperative hypotension. During the closure of the surgical site after deflating the tourniquet, we switched anesthetics from remimazolam to sevoflurane because the attending anesthesiologist had to leave for other duties. Then, repeated doses of phenylephrine were required throughout the remainder of the surgery, despite comparable surgical and other anesthetic conditions before and after the switch. After discontinuation of sevoflurane and remifentanil, noradrenaline was no longer required.

**Conclusions:**

This case report has shown that additional vasopressors were required during anesthesia maintenance with sevoflurane after switching from remimazolam.

## Background

Remimazolam besilate is a short-acting intravenous benzodiazepine approved for general anesthesia, which can be used for induction and maintenance of general anesthesia [1]. Compared with commonly used general anesthetics, such as sevoflurane and propofol, remimazolam has less suppressive effect on blood pressure in clinical settings [2–7]. During anesthesia maintenance, multiple factors can influence blood pressure, including patient characteristics (e.g., comorbidities, compensatory cardiovascular response to hypotension, and analgesic sensitivity), intravascular volume status, and administered drugs (e.g., vasoactive agents and analgesics). In clinical studies comparing the hemodynamic effects of anesthetics using interindividual comparisons, it is difficult to fully adjust for confounding factors. Intra-individual comparison can markedly reduce confounding, but its feasibility is limited in clinical settings.

We experienced a high-risk case in which we switched from remimazolam to sevoflurane for anesthesia maintenance during the surgical procedure involving closure of the surgical site with sufficient analgesia. The switch of anesthetics was performed because the attending anesthesiologist, who was experienced with remimazolam anesthesia, had to leave for other duties. This case allowed intra-individual comparison of the suppressive effect on blood pressure of remimazolam and sevoflurane.

## Case presentation

A 57-year-old woman (164 cm, 49 kg, American Society of Anesthesiologists physical status [ASA-PS] III) was scheduled to undergo amputation of her right lower leg due to diabetic foot gangrene. This patient had a history of diabetes, coronary artery stenosis, left carotid artery stenosis, right arteriosclerosis obliterans, and right brachiocephalic artery stenosis. The patient also had a history of recovering from septic shock a few months ago. Preoperative echocardiogram showed an ejection fraction of 64%, aortic calcification, and the mitral and tricuspid valves with mild regurgitation. A first-year senior resident of anesthesiology and an attending anesthesiologist were in charge. Standard monitoring included an electrocardiogram, pulse oximetry, and noninvasive blood pressure. After preoxygenation, anesthesia induction was performed with remifentanil at 0.05 μg/kg/min, remimazolam boluses totaling 13 mg (0.27 mg/kg; note that remimazolam boluses are off-label) followed by an infusion at 0.6 mg/kg/h, and rocuronium 50 mg. The airway was secured using a supraglottic airway device. Then, femoral, lateral femoral cutaneous, and sciatic nerve blocks were performed with 50 mL of 0.25% levobupivacaine. Nicorandil was administered at 4 mg/h throughout the surgery.

**Fig. 1:**
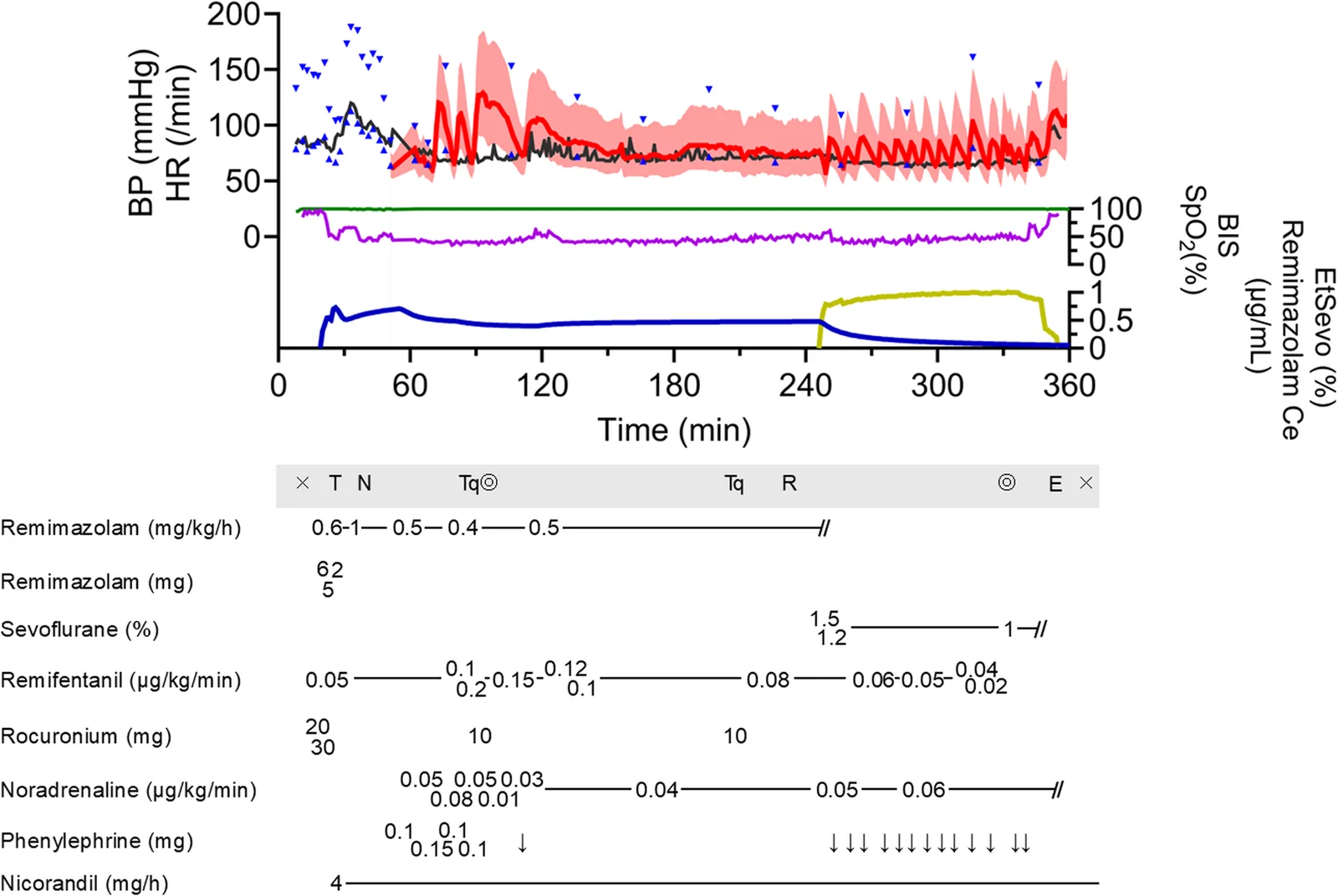
Vital signs, the Bispectral Index (BIS), effect-site Concentration (Ce) of remimazolam, and administered drugs. Pink band: invasive arterial pressure (systolic to diastolic range); Red line: mean invasive arterial pressure; Blue triangle: noninvasive arterial pressure (systolic and diastolic); Black line in the upper figure: heart rate (HR); Green line: peripheral capillary Oxygen saturation (SpO2); Purple line: BIS; Blue line: Remimazolam Ce; Yellow line: End-tidal Sevoflurane Concentration (EtSevo). Remark symbols in the gray band are as follows; ×: start and end of general anesthesia; T: insertion of a supraglottic device; N: peripheral nerve block; Tq: inflation and deflation of a tourniquet; ◎: start and end of surgery; R: specimen removal; E: removal of the supraglottic device; ↓: a bolus of phenylephrine 0.05 mg

The maintenance dose of remimazolam was initiated at 1 mg/kg/h after insertion of the airway device, then adjusted to 0.4–0.5 mg/kg/h during surgery (Fig. 1). To treat post-induction hypotension until the start of surgery, intermittent phenylephrine boluses were administered, followed by a continuous norepinephrine infusion at 0.01–0.08 µg/kg/min. After blood pressure stabilized, norepinephrine was infused at 0.03–0.04 µg/kg/min without additional vasopressor boluses during remimazolam anesthesia. The tourniquet was inflated for 118 min from the start of surgery. After blood pressure became stable, remifentanil was infused at a rate of ≤ 0.1 µg/kg/min.

Approximately 35 min after tourniquet deflation during the closure of the surgical site, the attending anesthesiologist had to leave the operating room for other duties. Because the senior resident lacked experience with remimazolam, the attending anesthesiologist switched from remimazolam at 0.5 mg/kg/h to sevoflurane at a vaporizer setting of 1.5%. From 3 min later, the vaporizer setting was maintained at 1.2% until the end of surgery.

Shortly after switching to sevoflurane (Fig. 1), blood pressure dropped significantly. Frequent phenylephrine boluses were administered to maintain a mean arterial pressure at ≥65 mmHg over the next 80 min with a noradrenaline infusion at 0.05–0.06 μg/kg/min until the end of surgery (328 min in Fig. 1). During the period from just before the switch of general anesthetics until the end of surgery, the surgical procedure involved closing the surgical site. The remifentanil infusion rate was 0.08 μg/kg/min, starting 20 min before the anesthetic switch and continuing for 40 min, then 0.06 μg/kg/min for 15 min, 0.05 μg/kg/min for 20 min, and finally 0.04 μg/kg/min. BIS scores were similar between remimazolam and sevoflurane anesthesia during maintenance of anesthesia. The procedure was completed uneventfully. Noradrenaline was discontinued at emergence from general anesthesia due to elevated blood pressure. The patient was discharged from the operating room after extubation, with confirmation of alertness, stable vital signs, and adequate pain control. On the first postoperative day, we confirmed that the patient had no intraoperative awareness or other anesthesia-related complications. The patient’s recovery was uneventful apart from phantom limb pain, and she was discharged on the 21st day after surgery.

## Discussion

This case report shows that repeated doses of phenylephrine were required during the maintenance of anesthesia with sevoflurane after switching from remimazolam (Fig. 1).

After switching to sevoflurane, blood pressure decreased immediately, and repeated doses of phenylephrine were required. Simultaneously, the noradrenaline infusion rate increased by up to 50% throughout sevoflurane administration, and the remifentanil infusion rate was gradually reduced to 0.04 μg/kg/min until the end of surgery. There was no significant change in surgical stimulation. A tourniquet was used during the amputation, but it was deflated 36 min before the anesthetic switch. Additionally, vital signs indicated that peripheral nerve blocks with opioid administration provided sufficient pain relief. These factors suggested that changes in anesthetic concentration were the primary cause of hemodynamic suppression. The intervals of phenylephrine boluses were similar between the first 30 min (average interval: 7.8 min) and the following 30 min (average interval: 6.5 min) despite a significant decrease in the effect-site concentration of remimazolam during the first 30 min (Fig. 1). The effect-site concentrations of remimazolam, which were calculated using the Masui model [8, 9] with TIVA trainer (https://www.tivatrainer.com/), at 0, 30, 60, and 90 min after the termination of remimazolam infusion were 0.48, 0.16, 0.10, and 0.07 μg/mL, respectively (Fig. 1). The time courses of end-tidal sevoflurane concentration and remimazolam effect-site concentrations, and the intervals of phenylephrine boluses suggested that sevoflurane was the primary cause of the decrease in blood pressure, and that remimazolam had little suppressive effect on hemodynamics. A previous article suggested that remimazolam can help prevent intraoperative hypotension by maintaining cardiac output and vascular resistance [10]. On the other hand, volatile anesthetics cause a drop in blood pressure by concentration-dependent reduction in myocardial contractility, vascular resistance, and preload [11].

The patient was expected to have an unstable hemodynamic status following anesthesia induction because she had multiple cardiovascular complications and was experiencing elevated levels of inflammation. Therefore, we selected remimazolam for anesthesia induction to reduce the incidence of post-induction and intraoperative hypotension. Although remimazolam was administered in three boluses (13 mg total) with a continuous infusion at 0.6 mg/kg/h for 6 min until placement of the supraglottic airway device, the lowest blood pressure was 104/67 mmHg before peripheral nerve blocks. A previous article suggests that remimazolam was associated with a lower incidence of PIH than propofol in ASA-PS III patients [12]. After administering 20 mL of 0.25% levobupivacaine for a peripheral nerve block, blood pressure decreased to 86/64 mmHg within 9 min, necessitating a vasopressor bolus. This occurred 4 min after a second 10 mL dose of the same medication for another peripheral nerve block. The decrease in blood pressure might be due to increased local anesthetic concentration [13], dehydration, the effects of remifentanil and remimazolam, or decreased sympathetic nervous system activity. During the nerve block, remimazolam Ce gradually increased from 0.60 to 0.71 µg/mL over 15 min, whereas remimazolam Ce for anesthesia maintenance was 0.45–0.48 µg/mL until the termination of remimazolam infusion.

There are strengths and limitations. This case report presents an intra-individual comparison of the hemodynamic effects of remimazolam and sevoflurane. Compared with interindividual comparisons, confounding factors would be limited. Because this is a case report of a high-risk patient, the differences in the hemodynamic effects of the two anesthetics may not be extrapolated, especially to a low-risk patient, whose compensatory response to hypotension would be better. In this case, the detailed hemodynamic changes, including cardiac function and vascular resistance, before and after the switch of anesthetics, are unclear. Another limitation was that the anesthetic switch in this case was single and unidirectional. Another case report or clinical trial examining hemodynamic changes following a switch from sevoflurane to remimazolam would help clarify the intraindividual impact of remimazolam.

This report demonstrates hemodynamic differences before and after switching anesthetics from remimazolam to sevoflurane, necessitating repeated phenylephrine doses to maintain adequate blood pressure in an ASA-PS III patient. These findings suggest that switching between remimazolam and an inhalational anesthetic may affect hemodynamic stability in hemodynamically unstable patients. The hemodynamic profile in this case is consistent with findings from previous studies [6, 7, 14–16], which indicate that remimazolam stabilizes intraoperative hemodynamics more effectively than sevoflurane.

## Data Availability

Not available.

## Abbreviations

BIS: Bispectral index
ASA-PS: American Society of Anesthesiologists physical status
